# Physiological and genomic characterization of *Arcobacter anaerophilus* IR-1 reveals new metabolic features in *Epsilonproteobacteria*

**DOI:** 10.3389/fmicb.2015.00987

**Published:** 2015-09-16

**Authors:** Irene Roalkvam, Karine Drønen, Runar Stokke, Frida L. Daae, Håkon Dahle, Ida H. Steen

**Affiliations:** ^1^Centre for Geobiology, University of BergenBergen, Norway; ^2^Department of Biology, University of BergenBergen, Norway; ^3^UniResearch, Centre for Integrated Petroleum ResearchBergen, Norway

**Keywords:** *Arcobacter*, *Epsilonproteobacteria*, genomics, metabolism, hox hydrogenase, ferric citrate reduction

## Abstract

In this study we characterized and sequenced the genome of *Arcobacter anaerophilus* strain IR-1 isolated from enrichment cultures used in nitrate-amended corrosion experiments. *A. anaerophilus* IR-1 could grow lithoautotrophically on hydrogen and hydrogen sulfide and lithoheterothrophically on thiosulfate and elemental sulfur. In addition, the strain grew organoheterotrophically on yeast extract, peptone, and various organic acids. We show for the first time that *Arcobacter* could grow on the complex organic substrate tryptone and oxidize acetate with elemental sulfur as electron acceptor. Electron acceptors utilized by most *Epsilonproteobacteria*, such as oxygen, nitrate, and sulfur, were also used by *A. anaerophilus* IR-1. Strain IR-1 was also uniquely able to use iron citrate as electron acceptor. Comparative genomics of the *Arcobacter* strains *A. butzleri* RM4018, *A. nitrofigilis* CI and *A. anaerophilus* IR-1 revealed that the free-living strains had a wider metabolic range and more genes in common compared to the pathogen strain. The presence of genes for NAD^+^-reducing hydrogenase (*hox*) and dissimilatory iron reduction (*fre*) were unique for *A. anaerophilus* IR-1 among *Epsilonproteobacteria*. Finally, the new strain had an incomplete denitrification pathway where the end product was nitrite, which is different from other *Arcobacter* strains where the end product is ammonia. Altogether, our study shows that traditional characterization in combination with a modern genomics approach can expand our knowledge on free-living *Arcobacter*, and that this complementary approach could also provide invaluable knowledge about the physiology and metabolic pathways in other *Epsilonproteobacteria* from various environments.

## Introduction

Free-living, environmental *Epsilonproteobacteria* are found to be ubiquitous in marine and terrestrial habitats, such as hydrothermal systems, marine sediments, pelagic seawater, acid mine drainage, lakes, springs, sulfidic caves and hydrocarbon-rich ground water (Campbell et al., 2006; Nakagawa and Takaki, 2009). They are in general associated with sulfide rich environments where they play a key role in the cycling of carbon, nitrogen, and sulfur (Campbell et al., 2006). *Epsilonproteobacteria* may also facilitate the colonization of other microbial groups due to detoxification of sulfur species, such as hydrogen sulfide (Campbell et al., 2006). Modern meta-omics technologies have revealed a wide distribution of free-living *Epsilonproteobacteria* (Huber et al., 2010; Akerman et al., 2013; Headd and Engel, 2014; Urich et al., 2014; Pop Ristova et al., 2015; Suh et al., 2015) and provided knowledge about their *in situ* metabolism (Dahle et al., 2013; Urich et al., 2014), however, there are relatively few cultivated representatives within this group. Isolates are obtained within the genera *Sulfurovum* (Inagaki et al., 2004; Mino et al., 2014), *Sulfurospirillum* (Finster et al., 1997; Stolz et al., 1999; Luijten et al., 2003; Kodama et al., 2007), *Nautiliales* (Alain et al., 2002; Takai et al., 2005; Grosche et al., 2015), *Nitratifractor* (Nakagawa et al., 2005b), *Sulfuricurvum* (Kodama and Watanabe, 2004), *Sulfurimonas* (Inagaki et al., 2003; Takai et al., 2006; Labrenz et al., 2013), *Thiomicrospira* (Brinkhoff et al., 1999; Knittel et al., 2005; Sorokin et al., 2006), *Thioreductor* (Nakagawa et al., 2005a) and *Arcobacter* (Donachie et al., 2005; Collado et al., 2009; Kim et al., 2010). Many of the free-living *Epsilonproteobacteria* associated with hydrothermal vents can perform oxidation of hydrogen or sulfur species (e.g., elemental sulfur, thiosulfate and hydrogen sulfide) with reduction of elemental sulfur, sulfite, thiosulfate, nitrate or low concentrations of oxygen (Alain et al., 2002; Miroshnichenko et al., 2002; Inagaki et al., 2003, 2004; Kodama and Watanabe, 2004; Nakagawa et al., 2005a, 2007; Mino et al., 2014). However, organoheterotrophic species within *Arcobacter* (McClung and Patriquin, 1980; Gevertz et al., 2000), *Thiomicrospira* (Takai et al., 2004) and *Sulfurospirillum* (Finster et al., 1997; Stolz et al., 1999; Luijten et al., 2003) have also been isolated from various environments. Of these, *Arcobacter* is the only genus with both pathogenic and free-living, non-pathogenic taxa. The first cultivated representatives of the genus *Arcobacter* were isolated from aborted bovine fetuses nearly 40 years ago (Ellis et al., 1977) and classified within the genus *Campylobacter*. As this genus includes many pathogens, the main focus of the characterization of first *Arcobacter* isolates was to distinguish between different species and survey the strains for resistance to antibiotics (Neill et al., 1985; Kiehlbauch et al., 1991; Vandamme et al., 1992). The genus *Arcobacter* was resolved by Vandamme et al. (1991), and since then several new species have been described, both pathogenic and free-living strains. The new species have been isolated from a remarkably broad range of habitats, such as humans and animals (Houf et al., 2005, 2009), marine environments (Wirsen et al., 2002; Kim et al., 2010), roots of estuarine salt march plant (McClung and Patriquin, 1980), sewage (Collado et al., 2011; Levican et al., 2013), shellfish (Collado et al., 2009; Figueras et al., 2011a,b; Levican et al., 2012), hypersaline environments (Teske et al., 1996; Donachie et al., 2005), estuarine sediments (Sasi Jyothsna et al., 2013), and even an oil field brine (Gevertz et al., 2000). The metabolic potential of most environmental free-living *Arcobacter* species has not been examined in detail. A lithoautotrophic lifestyle has only been described for the free-living *Arcobacter* strains FWKO B and CAB (Gevertz et al., 2000; Carlström et al., 2013), while the remaining strains are cultivated as organoheterotrophs. Furthermore, genomic information is available for a few *Arcobacter* species, and only one of the four available genomes originate from a free-living species; i.e., *A. nitrofigilis* CI (Pati et al., 2010).

Here we have isolated and characterized the novel strain IR-1 affiliated with *Arcobacter anaerophilus* originating from nitrate-amended corrosion experiments. The thorough growth experiments and genomic information revealed that the strain has several unique features relative to *Epsilonproteobacteria* in general and to *Arcobacter* in particular. The characterization showed that the IR-1 strain has a lithoautotrophic, lithoheterotrophic, or organoheterotrophic lifestyle coupled to a wide selection of electron acceptors. Genomic information from the IR-1 strain, the pathogenic *A. butzleri* RM4018 and free-living *A. nitrofigilis* CI showed that the free-living strains had more genes in common and in general a wider metabolic range than the pathogenic strain. We also extend the metabolic properties of *Epsilonproteobaceteria* by showing an ability to use iron-citrate as an electron acceptor, utilize tryptone, oxidize acetate with elemental sulfur and involve a *hox* hydrogenase in the central metabolism. Unique metabolic traits for *Arcobacter* were also revealed by strain IR-1, such as genes encoding nitrogen-fixation and an incomplete denitrification pathway, where nitrite is the end product from the reduction of nitrate.

## Materials and Methods

### Sampling Site and Isolation

The IR-1 strain was isolated from an enrichment culture grown on water from the Utsira Aquifer (UA) added 1:150 injection water (i.e., production water from Oilfield A and aquifer water mixed 1:1), a slice of sterile iron foil (20–50 mg, 0.1 mm, Alfa Aesar) and 12 mM S^o^ (Merck), as previously described (2014). The UA water is anaerobic and fully saturated with CH_4_ and CO_2_, saline (40%) and has a naturally low concentration of sulfate (0–5 mM; Drønen et al., 2014). The bottle was shaken at 100 rpm for 8 months at 25°C, before the remaining iron chips were harvested and stored at -80°C in enrichment medium added 15% glycerol. From here fresh enrichment cultures were initiated on Marine Broth 2216 added 5 mM NaNO_3_. The culture was transferred to agar plates of Marine Broth 2216 added 5 mM NaNO_3_ in anaerobic atmosphere (10% H_2_, 20% CO_2_, and 70% N_2_), and single colonies were transferred to new plates twice. Cells with a single morphology remained in the culture, and sequencing the 16S rRNA gene of the isolate using the primers 8f (5′-AGAGTTTGATCCTGGCTCAG-3′) (Edwards et al., 1989) and 1392r (5′-ACGGGCGGTGTGTRC-3′) (Lane et al., 1985) confirmed that the strain was 99% identical to *A. anaerophilus* strain JC83^T^ (Sasi Jyothsna et al., 2013), hence we named the new isolate *A. anaerophilus* IR-1.

### Cultivation

Growth medium for the isolate was based on an anaerobic mineral medium for nitrate reducers (NRB-medium) buffered with bicarbonate and supplied with trace element solution SL-10 and vitamins, as described by Myhr and Torsvik (2000). During experiments to determine optimal growth temperature, NaCl concentration and pH range; cultures were supplied with 30 mM acetate and 0.05% yeast extract as energy source and 8.5 mM nitrate as electron acceptor. For the pH range experiments, the medium was buffered with either 30 mM bicarbonate (Sigma–Aldrich) or 10 mM HEPES (Sigma). *A. anaerophilus IR-1* was transferred to fresh medium twice prior to the growth curve experiments. Optical density (OD) measurements were generated every 10–15 min at 600 nm, measured by the Cary 100 Bio UV/VIS-spectrophotometer (Varian) in 10 mm quartz glass cuvettes (Hellma). Instead of Gram staining, a KOH string test was performed as described by Ryu (1938). For a metabolic characterization of the isolate, NRB medium with 2% NaCl and pH of 7.2–7.3 was used. Substrates and e-acceptors were added according to **Table 2**. The closest relative, *A. anaerophilus* JC83^T^ (=DSM-24636), was obtained from the Deutsche Sammlung von Mikroorganismen und Zellkulturen (DSMZ) and included in the characterization as a reference strain.

Presence of enzymatic activity, such as urease, indoxyl acetate hydrolysis and oxidase, was investigated using Diatabs (Rosco Diagnostica) according to the manufacturer’s protocol. In addition, a catalase test was performed using 30% (w/w) hydrogen peroxide solution (Sigma) and colonies of *Escherichia coli* as positive control.

Nitrite formation was quantified using colorimetry, where the coloration generated by a nitrite test kit (Sera) was quantified in a Cary 100 Bio UV/VIS-spectrophotometer (Varian) at 550 nm. Here, *A. anaerophilus* IR-1 was cultivated on 10 mM acetate and 5 mM nitrate for 48 h at 35°C. The nitrite standard curve, negative control and parallel cultures of *A. anaerophilus* IR-1 were measured in duplicates.

### DNA Extraction and Sequencing

DNA was extracted from cells in parallel cultures of 30 ml NRB medium added 30 mM acetate and 8.5 mM nitrate. Cells were harvested by centrifugation at 5000 × *g* for 25 min, and the DNA extraction was based on the protocol described by Marmur (1961). In short: The pellet was dissolved in TE buffer (pH = 8) and 1% SDS, and the mix was incubated at 65°C for 5 min. Then perchlorate was added to a final concentration of 1 M and shaken well. The solution was added equal amounts of chloroform:isoamyl alcohol (24:1), followed by centrifugation at 5000 × *g* for 10 min. This step was repeated twice using only the aqueous phase. The nucleic acids were mixed 1:2 with 96% ethanol, incubated for 30 min on ice, and finally centrifuged at 13000 × *g* for 20 min at 4°C. The pellet was washed twice in 70% ethanol and dissolved in TE buffer (pH = 8). For the RNase treatment, the parallel DNA extractions were pooled. Ribonuclease A (Sigma) was added to a final concentration of 50 ng/μl, and the sample was incubated at 37°C for 30 min. One step of deproteinization with chloroform:isoamylalcohol (24:1) was performed as described above. The aqueous phase was added sodium acetate (pH = 5.2) to a final concentration of 0.3 M, and the DNA was precipitated and purified with ethanol. The DNA pellet was dissolved in 10 mM Tris buffer (pH = 8.0). A total of 35 μg high quality DNA (Ratio_A260/280_ = 1.98) was obtained and the DNA was sequenced at the Norwegian Sequencing Center for sequencing. A library was prepared using the Pacific Biosciences 10 kb library protocol, and size selection was done using BluePippin. The library was sequenced on a Pacific Biosciences RS II instrument using P4-C2 chemistry, where three SMRT cells were used in total.

In addition, DNA:DNA hybridization analysis of high quality DNA of *A. anaerophilus IR-1* and *A. anaerophilus* JC83^T^ was performed by DSMZ by renaturation rate measurements (De Ley et al., 1970), under consideration of the modifications described by Huss et al. (1983) using a model Cary 100 Bio UV/VIS-spectrophotometer equipped with a peltier-thermostatted 6 × 6 multicell changer and a temperature controller (Varian).

### Bioinformatics

Reads were assembled into contigs using the software Hierarchical Genome Assembly Process (HGAP) v2 (Chin et al., 2013) from Pacific Biosciences. The Prokka software (Seemann, 2014) and the RAST-server (Aziz et al., 2008; Overbeek et al., 2014) were used for automatic annotation of the genome. Predicted genes were also aligned to the NCBI database using a standalone BlastP search (Altschul et al., 1990). For identification of peptidases and adhesion-associated proteins encoded in the genome, the MEROPS peptidase database (Rawlings and Morton, 2008) and Pfam protein families database (Finn et al., 2014) were used. PacBio sequencing raw-data have been submitted to the Sequencing Read Archive (SRA) under BioProject PRJNA273926 and BioSample SAMN03316823.

Contigs of the assembled draft genome of *A. anaerophilus* IR-1 have been deposited as a WGS project in GenBank under the accession number JXXG01000000.

## Results

### Characterization of Isolate

The *A. anaerophilus* IR-1 strain was isolated from injection water in an enrichment culture added UA water, zerovalent iron, and elemental sulfur, which was used in corrosion monitoring (Drønen et al., 2014). The 16S rRNA gene of *A. anaerophilus IR-1* was 99% identical to *A. anaerophilus* strain JC83^T^ (Sasi Jyothsna et al., 2013), and the DNA:DNA hybridization analysis confirmed that *A. anaerophilus IR-1* is a new strain within *A. anaerophilus* with a 76.3–80.2% DNA:DNA hybridization value in comparison with *A. anaerophilus* strain JC83^T^.

**FIGURE 1:**
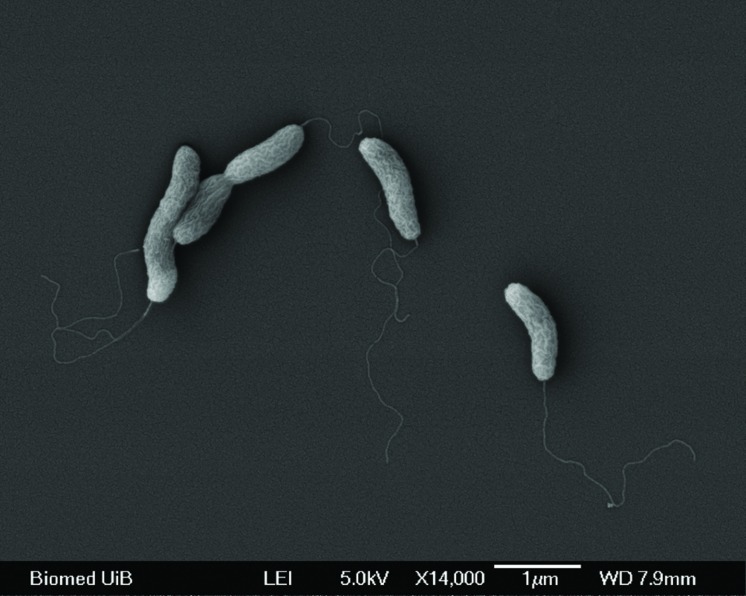
**Scanning electron micrographs of *Arcobacter anaerophilus* IR-1.** Cells are curved rods with a single polar flagellum.

The cells of the new strain were gram negative, curved rods, 1.5–2 μm long and 0.4–0.5 μm wide (**Figure 1**), and were observed as single cells or in chains of 2–6 cells during active growth. Cells were motile by a single polar flagellum (**Figure 1**), and were particularly active at early stages of growth. Cells were non-spore forming. The physiological characteristics of strain IR-1 were compared to published descriptions of other *Arcobacter* species (**Table 1**) using a selection of parameters devised to distinguish members of the family *Campylobacteraceae*, as resolved by the International Committee on Systematic Bacteriology (Ursing et al., 1994). Strain IR-1 grew under microaerophilic conditions at 37°C, but not at 42°C or under aerobic conditions. Growth was observed at both 0.5 and 4% NaCl. The strain did not utilize 1% glycine, and no hemolysis was observed. The strain was oxidase and urease positive, but did not have enzyme activity for catalase or indoxyl acetate hydrolysis. The new isolate showed a unique profile of characteristics among the chosen representatives of *Arcobacter*, and could be distinguished from other *Arcobacter* species based on three or more parameters (**Table 1**).

**Table 1:** Characteristics that differentiate *Arcobacter anaerophilus IR-1* from other strains within the genus *Arcobacter.*

Characteristics	1	2	3	4	5	6	7	8	9	10	11
Growth in/on											
Air at 37°C	-	-	-	+	+	+	ND	ND	+	ND	+
CO_2_ at 37°C^∗^	+	-	-	+	+	+	+	+	+	+	+
CO_2_ at 42°C^∗^	-	-	-	-	-	(+)^a^	+	-	(+)^a^	-	(+)^a^
0.5% (w/v) NaCl	+	+	+	-	-	+	+	+	+	+	ND
4% (w/v) NaCl	+	+	+	+	+	+	+^b^	-	-	-^b^	+
1% (w/v) glycine	-	+	-	+	-	ND	+	-	-	-	-
Hemolysis	-	-	-	-	-	-	V	+	-	-	-
Enzyme activity											
Oxidase	+	+	+	+	+	+	+	+	+	+	+
Catalase	-	-	+	-	-	(+)	(+)	+	(+)	+	+
Urease	+	-	+	-	-	-	-	-	+	-	-
Nitrate reduction	+	+	+	+	+	-	+	+	+	-	+
Indoxyl acetate hydrolysis	-	+	+	+	+	-	+	+	+	+	-

*1, Arcobacter anaerophilus IR-1 (This study); 2, A. anaerophilus JC83 DSM-24636^T^ (Sasi Jyothsna et al., 2013); 3, A. nitrofigilis DSM-7299^T^ (McClung and Patriquin, 1980; Sasi Jyothsna et al., 2013); 4, A. marinus DSM-24769^T^ (Kim et al., 2010; Sasi Jyothsna et al., 2013); 5, A. halophilus DSM-18005^T^ (Donachie et al., 2005); 6, A. mytilii DSM-23625^T^ (Collado et al., 2009); 7, A. butzleri DSM-8739^T^ (Vandamme et al., 1992); 8, A. skirrowii DSM-7302^T^ (Vandamme et al., 1992); 9, A. defluvii DSM-25359^T^ (Collado et al., 2011); 10, A. cryaerophilus DSM-7289^T^ (Vandamme et al., 1992); 11, A. molluscorum (Figueras et al., 2011a).*

*+, positive; –, negative; (+), weakly positive; V, variable among strains; ND, not determined.*

*^∗^Growth at microaerophilic conditions.*

*^a^At aerobic atmosphere.*

*^b^At 3.5% (w/v) NaCl.*

A more comprehensive characterization showed that the isolate could be cultivated under anaerobic or microaerophilic conditions. The temperature range was 15–40°C, with an optimum at 37°C. The salinity range was 0.5–6% NaCl, with an optimum at 2% NaCl. No growth was observed using 0% NaCl in the medium. The pH optimum was 7.5 in bicarbonate buffered medium, and pH 7.2 in the HEPES buffered medium. Carbon sources and rich complexes, such as acetate, lactate, peptone, pyruvate, tryptone, and yeast extract were utilized with oxygen or nitrate as electron acceptor (**Table 2**). Yeast extract, peptone and pyruvate were also fermented without an external electron acceptor. Inorganic compounds, such as hydrogen (H_2_), elemental sulfur, hydrogen sulfide, and thiosulfate could also be utilized if CO_2_ or small amounts of acetate (1 mM) was provided as carbon source (**Table 2**). Sugars were not utilized. In addition to respiration with oxygen at microaerophilic levels (3–10%); nitrate, elemental sulfur, and ferric citrate were also identified as electron acceptors. Ferric hydroxide and sulfate were not used as terminal acceptors. The *A. anaerophilus* IR-1 could be distinguished from its closest relative, *A. anaerophilus* JC83^T^, on the basis of lactic acid utilization and the capacity to hydrolyse indoxyl acetate (**Table 2**).

**Table 2:** Characteristics and substrate range of *A. anaerophilus IR-1* and the closest related species *A. anaerophilus* JC83^T^.

Characteristic	*A. anaerophilus* IR-1	*A. anaerophilus* JC83^T^
Cell size	1.5–2 μm long, 0.4–0.5 μm wide	1–2 μm long, 0.1–0.3μm wide^a^
Motility	+	+
DNA G + C content	30.2% mol	24.6% mol^a^
Genome size	3.26 Mbp	ND
Substrate utilization		
Acetate	+	+
Caproate	-	-
Cellobiose	-	-
Citrate	-	-
D-Fructose	-	-
D-Galactose	-	-
D-Glucose	-	-
DL-Lactate	+	-
Elemental sulfur	+^b^	+^b^
Ferrous (Fe^2+^) iron	-	-
Formate	-	-
H_2_	+	+
H_2_S	+	+
L-Arabinose	-	-
Peptone	+	+
Pyruvate	+	+
Sucrose	-	-
Thiosulfate	+^b^	+^b^
Tryptone	+	+
Yeast extract	+	+
Electron acceptors		
Nitrate	+	+
Elemental sulfur^c^	+	+
Sulfate	-	-
Thiosulfate	-	-
Ferric (Fe^3+^) citrate	+	+
3% O_2_	+	+
5% O_2_	+	+
10% O_2_	+	+
Air	-	-
Enzymatic reactions		
Urease	+	+
Indoxyl acetate hydrolysis	-	+
Oxidase	+	+
Catalase	-	-

*^a^(Sasi Jyothsna et al., 2013).*

*^b^Culture supplied with organic carbon source.*

*^c^Sulfide in the medium might have formed polysulfide from sulfur.*

### Genomic Information

Sequencing of the *A. anaerophilus* IR-1 genome generated 92 845 reads with an average read length of 6073 bp. The reads were assembled into seven contigs, comprising 3.257 Mbp in total. The size-distribution and sequencing coverage of assembled contigs suggested that three of the contigs constitute the chromosomal genome, while the remaining four could be extra chromosomal elements, such as plasmids. By using the Prokka annotation tool, 3596 protein coding genes were identified (Supplementary Table S1). The genome also contained 60 non-coding genes comprising four rDNA operons (Supplementary Table S2) localized on two different contigs, in addition to 48 tRNAs. The GC-ratio was 30.2% mol.

### Central Metabolism

From analyses of the *A. anaerophilus* IR-1 genome we identified a genotype that was in agreement with the phenotype revealed by the physiological characterization (**Table 3**). Presence of genes encoding energy converting Ni/Fe hydrogenase (*hydABC*), a Ni/Fe uptake hydrogenase (*hupSL*) and a cytoplasmic NAD^+^-reducing hydrogenase (*hoxEFHUY*) were congruent with the use of H_2_ as an electron donor. A complete SOX system (*soxABCDXYZ*) and sulfide:quinone oxidoreductase (*sqr*) was most likely involved in oxidation of hydrogen sulfide, thiosulfate, and elemental sulfur. The SOX system may also be involved in sulfite oxidation; however, sulfite was not tested as a substrate during the physiological characterization. Regarding central carbon metabolism, the genome encoded a complete TCA cycle, pentose phosphate pathway, Entner-Doudoroff pathway and glycolysis and gluconeogenesis, with the exception of genes encoding hexokinase/glucose 6-phosphatase. Also, genes encoding a reductive TCA cycle were the only identified genes associated with a CO_2_ fixation pathway. Key genes for utilization of lactate and pyruvate were found, including L-lactate dehydrogenase (*ldh*), pyruvate synthase (*por*) and phosphoenol pyruvate synthase (*pps*). Two pathways for acetate oxidation were encoded: a two-step reaction involving acetate kinase (*ack*) and phosphate acetyltransferase (*pta*) or a single step reaction involving acetyl coenzyme A synthetase [both the ADP dependent (*acd*) and AMP forming (*asc*) genes were found]. The acetyl-CoA formed could enter the TCA cycle by citrate synthase (*glt*) or malate synthase (*glc*) or be included in various anabolic pathways. The presence of formate dehydrogenase (*fdh*) genes indicates a potential for growth by formate oxidation, however, neither *A. anaerophilus* IR-1 nor *A. anaerophilus* JC83^T^ could grow on this substrate (**Table 2**).

**Table 3:** Comparison of genes involved in central metabolisms within selected species of *Arcobacter*, based on RAST annotations.

Pathway	*A. anaerophilus* IR-1	*A. nitrofigilis* CI	*A. butzleri* RM4018
Genome size (Mbp)	3.26	3.22	2.33
Hydrogenase			
NAD^+^-reducing	HoxEFHUY	-	-
Ni/Fe hydrogenase	HydABC	HydABC	HydABC
Ni/Fe uptake hydrogenase	HupSL	HupSL	HupSL
Sulfur oxidation			
Sox	SoxABCDXYZ	SoxABCDHXYZ	SoxABCDXYZ
Sulfide:quinone oxidoreductase	Sqr^∗^	Sqr^∗∗^	Sqr^∗∗^
Central carbon metabolism			
Glycolysis/Gluconeogenesis	+	+	+
Entner-Duorodorf pathway	+	+	-
Pentose phosphate way	+	+	+
Lactate dehydrogenase	+	+	-
Pyruvate:ferredoxin oxidoreductase	+	+	-
Phosphoenol pyruvate synthase (pps)	+	-	-
Acetyl coenzyme A synthetase	+	+	+
Malate synthase	+	-	-
TCA	+	+	+
rTCA	+	+	+
Nitrogen fixation			
Nitrogenase molybdenum-iron protein	NifDHK	NifDHK	-
Oxygen reduction			
NADH ubiquinone oxidoreductase	NuoABCDEFGHIJKLMN	NuoABCDEFGHIJKLMN	NuoABDEFGIJKLMN
Succinate degydrogenase	FrdABC	FrdABC	FrdABC
Cytochrome bc1	PetABC	PetABC	PetABC
Cytochrome c oxidoreductase	CcoNOP	CcoNOP	CcoNOP
Cytochrome d ubiquinol oxidase	CydAB	CydAB	CydAB
Nitrate reduction			
Nitrate reductase	NapABCDFGHL	NapABCDFGHL	NapABDFGHL
Nitrite reductase	-	NirBD	NrfAH
Nitric oxide reductase (nor)	-	-	NorB
Nitrous oxide reductase (nos)	NosZ	-	-
Sulfur reduction			
Polysulfide reductase	PsrAB	NrfD^∗∗^	-
Tetrathionate reductase	TtrABC	TtrABC	-
Anaerobic dimethyl sulfoxide reductase	DsmABC	-	-
Iron reduction			
Ferric reductase	Fre	-	-
Formate-dependent nitrite reductase	NrfD	-	-

*^∗^Present in dataset from Prokka.*

*^∗∗^Present in dataset from NCBI.*

As the new strain could utilize organic rich substrates like yeast extract, peptone and tryptone, a search in the MEROPS peptide database was performed in order to survey the genome for membrane associated peptidases that could initiate the degradation of complex protein molecules. In total, 80 different peptidases were identified, which were classified within the families: aspartic (4), cycteine (19), metallo (26), asparagine (2), serine (21), threonine (4) and unknown (4). Indications of protein-rich substrate as a preferable carbon source in *A. anaerophilus* IR-1 was strengthen by identification of genes encoding dipeptide chemoreceptor protein (*tap*) and dipeptide transport ATP-binding protein (*ddp*) involved in chemotaxis toward peptides, and membrane transport proteins, such as inner membrane amino-acid ABC transporter permease protein (*yecS, yhdGY*), oligopeptide transport system permease protein (*oppC*) and transporter permeases for leucine, isoleucine, valine, glutamine, arginine, glycine, and proline.

Two gene clusters encoding nitrogenase (*nifDHK*) were identified in the genome of strain IR-1 (**Table 3**), where one gene cluster is also present in *A. nitrofigilis*. These genes have previously not been described for members of the genus *Arcobacter*, however, our results suggest that free-living strains within this genus can sustain themselves with a nitrogen source by converting dinitrogen to ammonia.

### Respiration

As expected, genes for aerobic respiration were found in the genome of the microaerophilic *A. anaerophilus* IR-1. Genes encoding complex I–IV of the respiratory chain was present, including NADH ubiquinone oxidoreductase (*nuoABCDEFGHIJKLMN*; complex I), succinate dehydrogenase (*frdABC*; complex II), cytochrome bc1 complex (*petABC*; complex III) and cytochrome c oxidoreductase (*ccoNOP*; complexIV; **Table 3**). In addition, genes for cytochrome d ubiquinol oxidase (*cydAB*) were found (complex IV). The respiratory chain is linked to a F_0_F_1_ ATPase that generates ATP, and genes (*atpABCDEFGH*) encoding both domains were found. The *Arcobacter* strain IR-1 could also respire with nitrate to form nitrite, and accordingly, nitrate reductase (*napABCDFGHL*) genes were present in the genome (**Table 3**). However, the denitrification pathway was incomplete, where genes encoding nitrite reductase (*nir/nrf*) and nitric oxide reductase (*nor*) were lacking, while genes encoding nitrous oxide reductase (*nos*) were identified (**Table 3**). Congruent with the genomic information, a colorimetric method revealed an average nitrite concentration of 0.22 mM after 48 h in cultures grown on acetate and nitrate. Furthermore, cultures of *A. anaerophilus* IR-1 grew well in medium supplemented with elemental sulfur as terminal electron acceptor. However, the strain may also have benefitted from polysulfide that might have formed chemically in the medium since hydrogen sulfide was used as reducing agent. Polysulfide reductase genes (*psrAB*) were identified in the genome, suggesting a role for reduction of polysulfide to sulfide. Genes for tetrathionate reductase (*ttrABC*) were also found, which could form thiosulfate from tetrathionate, but genes for thiosulfate reductase were missing. Observation of anaerobic dimethyl sulfoxide (DMSO) reductase genes (*dsmABC*) in the genome indicated that DMSO could be a possible electron acceptor, but his was never tested *in vitro*. Genes for dissimilatory sulfate reduction were not found. The genomic survey also revealed a putative ferric iron reductase (*fre*), which could support the observed respiration with ferric citrate by the new isolate.

### Comparative Genomics

**FIGURE 2:**
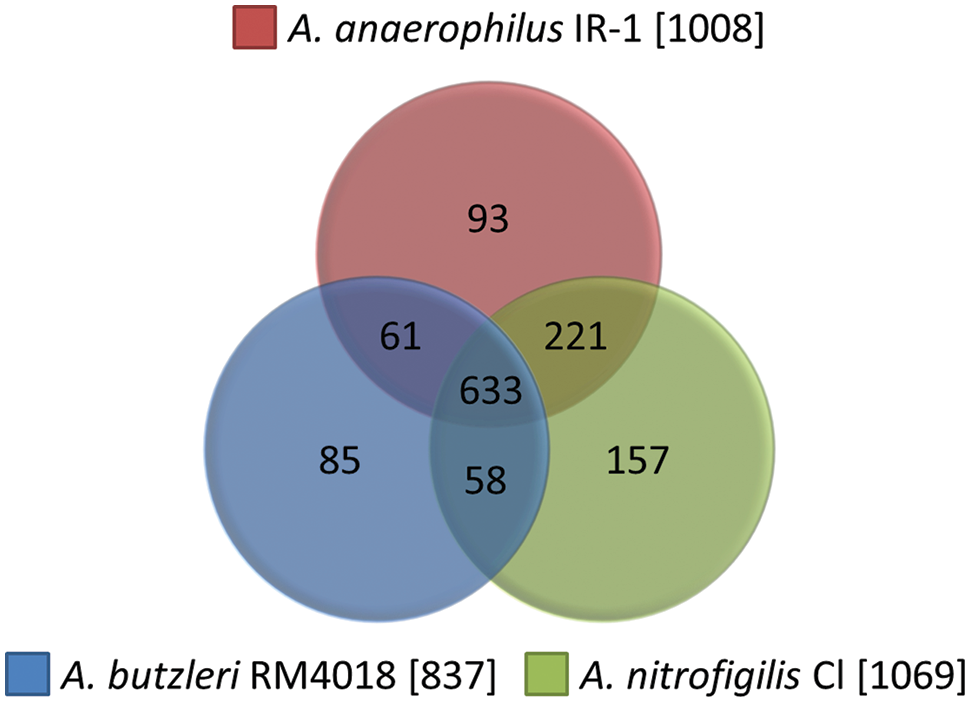
**Comparative genomics.** Unique genes from *A. anaerophilus* IR-1 (1008), *A. nitrofigilis* CI (1069), and *A. butzleri* RM4018 (837) annotated in RAST was extracted and compared.

The genome of *A. anaerophilus* IR-1 was compared to the free-living *A. nitrofigilis* CI (DSM 7299; Pati et al., 2010) and the pathogen *A. butzleri* RM4018 (DSM 8739; Miller et al., 2007). Genomes of *A. nitrofigilis* (NC_014166) and *A. butzleri* (NC_009850) were obtained from NCBI and uploaded in RAST for comparison with *A. anaerophilus* IR-1. The genome analyses of *A. anaerophilus* (3.26 Mbp) resulted in 1432 annotated genes in RAST, while 1542, and 1244 annotated genes, respectively, were identified in *A. nitrofigilis* (3.22 Mbp) and *A. butzleri* (2.33 Mbp). Duplicate genes in each genome were removed from the dataset; leaving *A. anaerophilus*, *A. nitrofigilis*, and *A. butzleri* with 1008, 1069, and 837 unique genes, respectively; of which 633 genes were common for all three genomes (**Figure 2**). The genomes of free-living *A. anaerophilus* and *A. nitrofigilis* showed highest similarity, with 221 shared genes, while the pathogen *A. butzleri* had around 60 genes in common with the two free-living species (**Figure 2**). The genomes also comprised genes that were unique for each specimen: *A. anaerophilus* (93), *A. nitrofigilis* (157), and *A. butzleri* (85).

The function based comparison tool in RAST and searches in the Prokka dataset (*A. anaerophilus*) or the genome dataset from NCBI (*A. nitrofigilis* and *A. butzleri*) were used for identifying key genes in central carbon metabolism, sulfur oxidation, hydrogen oxidation, and respiration with oxygen, nitrate, sulfur, and iron; thereby providing genomic comparisons of the metabolic capacity based on selected pathways (**Table 3**). This approach revealed that *A. anaerophilus* was the most metabolically versatile species, with unique genes involved in hydrogen oxidation (*hox*), nitrate reduction (*nosZ*), reduction of sulfur species (*psr, dsm*), iron reduction (*fre*), in addition to central carbon metabolism, such as phosphoenol pyruvate synthase and malate synthase (**Table 3**). Furthermore, the free-living strains were sharing some metabolic properties that seemed to be absent in *A. butzleri*, such as genes for nitrogen fixation (*nif*), the *napC* subunit in the nitrate reductase complex, tetrathionate reduction (*ttr*) and several pathways in central carbon metabolism. Interestingly, *A. anaerophilus* IR-1 was lacking genes encoding a nitrite reductase, while nitrite reductase (*nir*/*nrf*) genes were found in the other two *Arcobacter species*. This indicates that both *A. nitrofigilis* and *A. butzleri* could have a mechanism for nitrite detoxification, which is not found in *A. anaerophilus*. The *A. butzleri* genome also comprised nitric oxide reductase *(norB)*, which was not present in the other species.

## Discussion

### New Metabolic Properties of Free-Living *Arcobacter*

Today there are 20 acknowledged species within the genus *Arcobacter*, including free-living strains and pathogens isolated from mammal and human sources. The genus is dominated by free-living strains isolated from various environments; however, knowledge about the metabolic capacity of these remains incomplete. In this study, extensive cultivation experiments with *A. anaerophilus* IR-1 revealed a versatile metabolism, including lithoautotrophy, lithoheterotrophy, and organoheterotrophy (**Table 2**) that was confirmed by genome analysis. A lithoautotrophic lifestyle has previously only been described for the free-living *Arcobacter* strains FWKO B and CAB (Gevertz et al., 2000; Carlström et al., 2013), however, this capability might be more widespread among the members of *Arcobacter* than anticipated, as most free-living species were never grown lithoautotrophically when characterized. During lithoautotrophic and lithoheterotrophic growth of *A. anaerophilus* IR-1, elemental sulfur, hydrogen sulfide, thiosulfate or H_2_ were used as electron donors in combination with organic molecules or CO_2_ as carbon source. This metabolic profile is similar to those of the free-living *Epsilonproteobacteria* of the genera *Nautiliales*, *Sulfurovum*, and *Sulfurimonas* isolated from hydrothermal vents and marine sediments (Alain et al., 2002; Miroshnichenko et al., 2002; Inagaki et al., 2003, 2004; Takai et al., 2006; Cai et al., 2014; Mino et al., 2014). The hydrogen sulfide may be oxidized autotrophically in *A. anaerophilus* IR-1 by *sqr* (**Table 3**). The presence of *sqr* was unique for this *Arcobacter* strain, but transcriptomic studies of microbial mats from hydrothermal vents and sediments have shown that the gene is highly expressed in free-living *Epsilonproteobacteria* (Dahle et al., 2013; Urich et al., 2014). Oxidation of elemental sulfur and thiosulfate, which required an organic carbon source, may be catalyzed by the SOX system (*soxABCDXYZ*) using the previously described mechanism (Friedrich et al., 2001; Bagchi and Ghosh, 2005; Dahl et al., 2008). The genes encoding the SOX system was found in all three *Arcobacter* genomes, which fits well with the metabolic traits of *Epsilonproteobacteria* in general. The Ni/Fe hydrogenase (*hyd*) used for hydrogen uptake was found in the *Arcobacter* genomes investigated in this study and is also found in other epsilonproteobacterial genera, such as *Campylobacter*, *Helicobacter*, *Sulfurimonas*, *Wolinella* (Dross et al., 1992; Benoit and Maier, 2008; Cordwell et al., 2008; Sievert et al., 2008). On the contrary, the NAD^+^-reducing hydrogenase (*hox*) seemed unique for *A. anaerophilus* IR-1 among *Arcobacter* and even among *Epsilonproteobacteria* (**Table 3**). The NAD^+^-reducing hydrogenase is a cytoplasmic, oxygen tolerant, bidirectional hydrogenase that has previously been identified in *Cyanobacteria* (Papen et al., 1986; Tamagnini et al., 2002), *Gammaproteobacteria* (Coppi, 2005), *Betaproteobacteria* (Burgdorf et al., 2005), *Actinobacteria* (Grzeszik et al., 1997) and phototrophic bacteria (Rákhely et al., 2004; Long et al., 2007). The exact function of this hydrogenase is not well understood, however, it has been suggested to play a role in removing excess electrons from fermentation or photosynthesis in *Cyanobacteria* and phototrophic bacteria, or supply complex I with reducing compounds (NADH) in aerobic bacteria in order to maintain a proton motive force (Horch et al., 2012). The NADH could also be converted to NADPH in the cytoplasm, hence the NAD^+^-reducing hydrogenase could provide electron donors for CO_2_ fixation in some bacteria. In *A. anaerophilus* IR-1, this hydrogenase may thus be involved in regulation of the NADH levels in the cells or possibly in NADPH generation for CO_2_ fixation. An organoheterotrophic lifestyle was also verified for the *A. anaerophilus* strains. To our knowledge, this is the first time a free-living *Arcobacter* has been shown to degrade tryptone with nitrate as electron acceptor, while degradation of yeast extract and peptone is also described for *Arcobacter* sp. CAB (Carlström et al., 2013). Utilization of smaller organic molecules, such as acetate, lactate, and pyruvate, seems to be a common trait within *Arcobacter*, and has been described for several species (McClung and Patriquin, 1980; Teske et al., 1996; Carlström et al., 2013; Sasi Jyothsna et al., 2013), including strain IR-1. Interestingly, *A. anaerophilus* JC83^T^ was unable to utilize lactate. The observed growth upon acetate with sulfur as the terminal electron acceptor by *A. anaerophilus* IR-1 has previously not been observed among *Epsilonproteobacteria*, and is so far only described for strains of *Desulfuromonas* and *Desulfurella* within *Deltaproteobacteria* (Pfennig and Biebl, 1976; Bonch-Osmolovskaya et al., 1990). Formate did not support growth of *A. anaerophilus* IR-1 or *A. anaerophilus* JC83^T^ in our growth experiments, although growth on formate is previously observed in *Arcobacter* sp. (Teske et al., 1996; Gevertz et al., 2000). The fdh in *E. coli* is oxygen sensitive, and the *fdhF* gene is induced by increasing formate concentrations and repressed by nitrate (Pecher et al., 1983; Wang and Gunsalus, 2003). The lack of growth of *A. anaerophilus* IR-1 on formate when oxygen or nitrate was provided as electron acceptor may be explained by a similar mechanism as in *E. coli.*

With a few exceptions, free-living *Arcobacter* are described as microaerophilic and nitrate reducing bacteria (Gevertz et al., 2000; Donachie et al., 2005; Collado et al., 2009, 2011; Kim et al., 2010; Figueras et al., 2011a,b; Levican et al., 2012, 2013; Sasi Jyothsna et al., 2013); and even elemental sulfur has been reported as electron acceptor (Gevertz et al., 2000). We observed that the *A. anaerophilus* strain IR-1 could use all of these during growth on organic or inorganic compounds (**Table 2**). The genome analysis suggests that complex IV in the electron transport chain comprise cytochrome c oxidoreductase or cytochrome d ubiquinol oxidase when oxygen is provided as terminal electron acceptor. In the betaproteobacterial *Azoarcus* sp. BH72, a cytochrome c oxidase and a quinol oxidase are expressed, however, they are upregulated according to oxygen concentrations where the former dominate during aerobic conditions and the latter during microaerophilic conditions (Reinhold-Hurek and Zhulin, 1997). In *E. coli*, the cytochrome d containing enzyme complex has a higher affinity for oxygen (Miller and Gennis, 1983; Kita et al., 1984), and this can thus indicate that this terminal oxidase may operate at low oxygen concentrations in *A. anaerophilus* IR-1. The cytochrome c oxidoreductase may operate at higher oxygen concentration, but still within the range of microaerophilic conditions.

All three *Arcobacter* strains included in the genome comparison have the capability of nitrate reduction (McClung and Patriquin, 1980; Miller et al., 2007), which is also recognized as a common property among other epsilonproteobacterial taxa (Vetriani et al., 2014). Genome sequencing of free-living *Campylobacter, Nitratiruptor, Sulfurimonas, Sulfurovum*, and *Wolinella* has revealed a complete denitrification pathway or nitrite ammonification pathway, meaning that nitrate can be reduced to dinitrogen or ammonium (Voordeckers et al., 2005; Takai et al., 2006; Nakagawa et al., 2007; Kern and Simon, 2009; Sikorski et al., 2010; Grote et al., 2012). The periplasmic enzyme nitrite reductase (*Nap*) is catalyzing the nitrate reduction to nitrite, and the nap operon (*napAGHBFLD*) in *A. anaerophilus* IR-1, *A. nitrofigilis*, and *A. butzleri* had the same gene orientation as commonly found in epsilonproteobacterial species (Sparacino-Watkins et al., 2014). In these species, electrons are transferred from the menaquinone pool to the terminal reductase (NapAB) via a ferredoxin containing integral membrane protein (NapGH; Kern and Simon, 2009; Sparacino-Watkins et al., 2014). However, the free-living strains within *Arcobacter* might also have the transmembrane protein NapC as an alternative electron transition pathway to NapAB, as genes encoding NapC were found downstream the *nap* operon in both *A. anaerophilus* and *A. nitrofigilis* (**Table 3**). This could provide the *Arcobacter* strains with a more advanced nitrate reductase complex that has previously only been seen in *Gammaproteobacteria* (Sparacino-Watkins et al., 2014). However, further studies are needed to confirm a function in nitrate reduction by NapC in free-living *Arcobacter* strains. The remaining denitrification pathway in *A. anaerophilus* IR-1 was incomplete, where only the genes encoding *nos* were identified (**Table 3**), indicating that nitrite produced by nitrate reductase may represent a metabolic end product. These findings were also supported by the detection of nitrite (0.22 mM) in cultures supplied with nitrate as electron acceptor. In comparison, *A. nitrofigilis* and *A. butzleri* encode nitrite reductase [NAD(P)H] (*nirBD*) and cytochrome C nitrite reductase (*nrfAH*), respectively (**Table 3**), which catalyzes the reduction of nitrite to ammonia. The periplasmic nitrite reductase in *A. butzleri* has probably direct supply of nitrite produced by nitrate reductase; however, the nitrite reductase in *A. nitrofigilis* is a cytoplasmic enzyme. Here, expression of the nitrite transporter gene found in the same gene cluster as the nitrite reductase might be involved in shuttling the nitrite over the membrane to the cytoplasmic enzyme complex. In the epsilonproteobacterial *Nautilia profundicola*, a different mechanism of nitrite removal is known, where nitrite is used in an assimilatory nitrogen pathways via hydroxylamine (Campbell et al., 2009). Genes encoding a similar pathway were not found in the genome of *A. anaerophilus* IR-1. Instead, the new *Arcobacter* strain seems to tolerate increasing nitrite concentrations (up to 0.3 mM in batch cultures).

In *A. anaerophilus* IR-1 a gene cluster comprising *nosZDFLY* and *napGH* was found, which is conserved in *Wolinella succinogenes* (Simon et al., 2004), while the gene cluster was lacking in *A. nitrofigilis* and *A. butzleri*. This gene cluster in the IR-1 strain also included two hypothetical genes that are annotated as cytochrome c in *W. succinogenes*. This gene cluster is also widely conserved among the *Epsilonproteobacteria*, and seems to be a unique characteristic of this bacterial class (Kern and Simon, 2009). The encoded proteins provide a link between the reduction of nitrous oxide to N_2_, catalyzed by NosZ, and electron transfer from the menaquinol pool by NapGH and cytochrome c. However, the metabolic importance of nitrous oxide reduction is not well studied for most *Epsilonproteobacteria* and the role in *A. anaerophilus* IR-1 metabolism remains to be identified.

The *A. anaerophilus* IR-1 strain was also found to reduce ferric iron with acetate as electron donor. The genome analysis suggests that a NrfD protein coupled to ferric reductase takes part in this reaction; a mechanism equivalent to the one proposed for *Melioribacter roseus*, where putative ferric reductase (*fre*) in the outer membrane is coupled to a NrfCD protein located in the inner membrane via a c-type cytochrome shuttle (Kadnikov et al., 2013). In *A. anaerophilus* IR-1, the NrfD protein was identified as a membrane bound formate-dependent nitrite reductase (Acr_01494) in the original Prokka annotation list. However, a standalone BlastP search using a 420 aa query sequence resulted in best hit against polysulfide reductase (*Sulfurospirillum arcachonese*) with a bit-score of 593 and 72% identity. Although the identity of the protein was difficult to determine, the protein was classified within the NrfD superfamily, which is involved in electron transfer from the quinone pool to a periplasmic receptor. Growth experiments showed that *A. anaerophilus* IR-1 could only reduce the soluble ferric citrate, which indicates that the strain requires chelated iron compounds for iron reduction. The insoluble ferric hydroxide was not utilized, and genes encoding conductive pili homologous to the nanowires in *Geobacter* (Reguera et al., 2005) were not found. Ferric iron minerals are abundant in anaerobic aquifers and sediments (Nealson, 1997; Orcutt et al., 2011), and humic substances have been shown to stimulate iron reduction in these environments where the humic substances serve as an electron shuttle between microorganisms and minerals (Lovley et al., 1996; Nevin and Lovley, 2000). However, this was not tested *in vitro* for strain IR-1. Overall, *A. anaerophilus* IR-1 could possibly reduce chelated iron minerals in a natural environment, and this ability expands the ecological niche of the free-living *Epsilonproteobacteria* to take part in cycling of iron.

## Conclusion

Through physiological characterization and genomics of *A. anaerophilus* IR-1, new knowledge about the metabolic properties of free-living *Epsilonproteobacteria* has been achieved, such as utilization of the complex organic substrate tryptone, reduction of ferric iron citrate and presence of genes encoding a NAD^+^-reducing hydrogenase (Hox). Comparative genomics of three *Arcobacter* species showed that the free-living strains had more in common compared to the pathogen strain. Their metabolic range of lithoautotrophy and organoheterotrophy is comparable to many free-living *Epsilonproteobacteria* isolated from hydrothermal vents and marine sediments. All the *Arcobacter* strains had an incomplete denitrification pathway; hence we suggest that members of the genus *Arcobacter* are nitrate reducers rather than denitrifiers. *A. anaerophilus* IR-1 did not have a mechanism for nitrite removal, and therefore seems to tolerate high concentrations of nitrite, while the other *Arcobacter* species encodes genes for nitrite reduction to ammonia.

## Conflict of Interest Statement

The authors declare that the research was conducted in the absence of any commercial or financial relationships that could be construed as a potential conflict of interest.
